# Outcomes after surgical repair of primary parastomal hernia

**DOI:** 10.1007/s10029-025-03267-1

**Published:** 2025-01-23

**Authors:** Nulvin Djebbara-Bozo, Nellie B. Zinther, Anette Søgaard, Hans Friis-Andersen

**Affiliations:** 1 Department of Breast and Plastic Surgery, Aalborg University Hospital, Søndre Skovvej 3, Aalborg, 9000 Denmark; 2 Department of General Surgery, Horsens Regional Hospital, Horsens, Denmark

**Keywords:** Parastomal hernia, Ostomy, Recurrence, Hernia correcting surgery, Hernia repair

## Abstract

**Purpose:**

Parastomal hernia is a frequent complication after stoma construction, with increasing incidence over time. Surgical repair is reported with a high recurrence rate and the evidence on the topic is limited. We conducted a retrospective study to evaluate the incidence of recurrence after parastomal hernia repair and assessed the risk factors and predictors for recurrence at the Regional Hernia Center at Horsens Regional Hospital, Denmark.

**Methods:**

119 patients underwent primary parastomal hernia repair from January 2017 until April 2021. Mean follow-up period was 72 months. Information including demographic data, non-modifiable risk factors and modifiable risk factors were assessed and analyzed using LASSO to select relevant predictors and GLM was employed hereafter.

**Results:**

Multivariate analysis showed that age, diabetes, IBD, constipation, and fecal incontinence were strong pre-operative predictors, with age, IBD, ileostomy, and colorectal cancer also reaching significance in univariate analyses. Post-operatively, EHS classification 1, and Clavien Dindo Grade 3b were identified as strong predictors in univariate analyses.

**Conclusion:**

Recurrence after parastomal hernia repair was 17.64% during a follow-up period of minimum 3.5 years.

## Introduction

Parastomal bulging is a common and feared complication after stoma construction. It is caused by an abnormal protrusion of the stoma or contents from the abdominal cavity through an abdominal wall defect related to the stomal orifice through the fascia. Numerous parastomal hernias (PSH) are asymptomatic, however, it is often necessary to repair due to a history of incarceration, obstruction, pain, leakage from ostomy and growing protrusion [1] leading to a decrease in the quality of life [2–4]. Despite prophylactic attempts to avoid PSH, the incidence remains high. Depending on the stoma, e.g., colostomy, ileostomy, urostomy and the length of follow-up, the incidence is reported up to 78% [3, 5, 6].

Different surgical techniques for PSH repair include laparoscopic and open approaches with or without prophylactic prosthetic mesh, local fixation or relocation of the stoma. Some centers suggest watchful waiting, due to the high risk and recurrence after PSH repair. According to different reports, up to 55% of the patients experience recurrence of their hernia after surgical repair [7–11]. As there is a lack of consistent definition of diagnosis and great variation in surgical approach in former studies, the literature is difficult to compare. Meanwhile these factors are also highly dependent on the specific surgeon evaluating the patient. Their judgment depends on their subjective experience and volume of surgical PSH repairs. Although there are older and also recently updated international guidelines regarding PSH repair, the evidence is weak and limits its use [12–15].

As a solution to the poor outcomes of recurrence after hernia repair, Denmark has centralized the surgical treatment of PSHs to five specialized hernia centers, which has led to significantly improved outcomes [16]. In the recent years initiatives have been established and standardized at the Regional Hernia Center in Horsens to enhance patient outcomes and reduce recurrence rates. Among these initiatives is the avoidance of emergency hernia reconstruction. Consequently, in cases of irreducible hernias, the emergent priority is to reduce the hernia, optimize the patient’s condition, and subsequently schedule either same-day surgery or later elective surgery.

The primary aim of this study was to review the recurrence after parastomal hernia repair after nationwide centralization. Secondly, to review outcomes of patients undergoing PSH repair, including identification of risk factors of recurrence.

## Methods

### Study design

This is a retrospective, descriptive study on surgical treatment of PSH surgery in Horsens Regional Hospital, which is one of the five specialized hernia centers in Denmark. All surgical procedures are registered in the electronic patient journal system (EPJ) and coded according to the International Statistical Classification of Disease (ICD-10). All patients undergoing PSH repair during January 2017 till March 2021 were identified by retrieving procedure codes regarding PSH correcting surgery from the electronic patient system, EPJ. As this is a quality study, our application only allowed us to go back five years in the medical records, thus limiting us in retrieving data on all the patients located in the register. We identified 119 patients, and their data were reviewed by one of the authors. The study included patients who underwent both urgent and elective parastomal hernia repair. Inclusion criteria were age over 18 and no former parastomal hernia repair. Sex, age, body mass index (BMI), smoking status and comorbidities were obtained. Details regarding the PSH and the PSH correcting surgery was registered along with postoperative events including Clavien-Dindo score, postoperative surgical complications, recurrence, and time of recurrence (see Table 1 for further details). PSH recurrence is defined as PSH detected following primary repair through either clinical assessment by a surgeon or imaging. If no event regarding PSH were to be found in the records, this was regarded as no recurrence. Follow up time was a mean of 72 months and SD of 15.2 months (see Table 1).

### Statistical analyses

In addition to univariate chi-square analyses (Monte Carlo simulations for small sample sizes), we also performed a multivariate analysis that shows a fuller picture by accounting for several variables at once. The univariate analysis, which is subject to disproportionate influence, is thus corrected for by using the multivariate approach.

We distinguish between modifiable and non-modifiable risk factors. Non-modifiable risk factors refer to variables that are pre-existing to a patient’s health or condition before any medical intervention. These are pre-operative factors inherent to the patient, such as sex, age, smoking status, co-morbidity, BMI, ASA classification, type of stoma, EHS classification, urgency of repair, reason for stoma and reason for PSH repair (see Table 2). However, modifiable risk factors refer to factors that are related to, or directly caused by, the surgical intervention. These are post-operative surgical complications, type of repair, Clavien Dindo index score, and time of recurrence (see Table 3). In separating modifiable from non-modifiable risk factors, we further decided to compute two separate generalized linear models (GLMs) using R Studio (V. 2023.03.0). Following statistical 2-step recommendations in variable selection [17], we first identified candidate variables based on existing literature and expert knowledge. We then subjected the selected variables to Least Absolute Shrinkage and Selection Operator (LASSO from “glmnet”) as recommended by Heinze et al. [18]. LASSO is a form of linear regression that selects variables and regularizes by adding an L1 penalty. This is to minimize residuals, which effectively simplify models by driving some coefficients to zero, for automatic feature selection and better interpretability. The selected candidate variables were further fitted to a GLM, which we report on below (Table 4). The same approach was used for modifiable risk factors. Reasonable outliers were excluded based on visual inspection of a normal Q-Q plot.

## Results

### Demographic overview

The overview in Table 1 provides detailed patient characteristics and composition. Patients ranged in age from 31 to 94 years, with an average of 68.1 years, where the majority (~ 83%) were non-smokers. Gender distribution was relatively equal, with approximately 41% male and 59% female. While about ~ 62% had no comorbidity, about 13.5% had either diabetes mellitus (DM) type 1 or 2. Additionally, the average BMI was 27.14 indicating an over-weight average patient group. Colostomy (~ 60%) and ileostomy (~ 30%) were the most frequent types of stomas, where colorectal cancer (~ 42%) and inflammatory bowel disease (IBD) (~ 19%) where the most frequent causes. The two most frequent reasons for hernia correcting surgery were intestinal obstruction (~ 27%) and pain related to ostomy (~ 26%). The most frequent type of repair was the Sugarbaker procedure (~ 61%). Our data contains 2/3 elective surgeries, leaving 1/3 for acute surgery. We have an over-representation of patients scoring Grade 1 in Clavien Dindo (~ 82%) compared to the other grades. While 57% experienced no post-operative complication, ~ 18% reported seroma post-surgery. Overall, we have a recurrence rate of 17.64%.

**Table 1:** Data overview

Sex ratio (Male/Female)	49/70	
**Age (years): x̄**,** σ [range]**	68.1 ± 12.7 [31; 94]	
**Follow-up (months): x̄**,** σ [range]**	72 ± 15.2 [42.1; 98.2]	
**Smokers**		
Non-smoker Current smoker Not stated	99 (83.19%) 15 (12.61) 5 (4.20)	119
**Co-morbidity**		
DM type 1 and 2 COPD Asthma	16 (13.45%) 20 (16.81%) 9 (7.56%)	119
No comorbidity	74 (62.18%)	
**BMI x̄**,** σ [range]**	27.14 ± 4.53 [18; 46]	
**ASA Classification**		
1 2 3 4 5	4 (3.36%) 69 (57.98%) 43 (36.13%) 3 (2.52%) 0 (0,00%)	119
**Type of stoma**		
Colostomy Ileostomy Loop ileostomy Urological ostomy	71 (59.66%) 35 (29.41%) 2 (1.68%) 11 (9.24%)	119
**Reason for stoma**		
Colorectal cancer Inflammatory bowel disease Benign sigmoid stenosis Intestinal ischemia Anal and rectal injuries Fecal incontinence Urological cancer Gynecologic cancer Perforated diverticulitis FAP Constipation	50 (42.02%) 23 (19.32%) 5 (4.2%) 3 (2.52%) 5 (4.2%) 9 (7.56%) 10 (8.40%) 1 (0.84%) 5 (4.20%) 1 (0.84%) 7 (5.88%)	119
**Reason for PSH correcting surgery**		
Intestinal obstruction Recurrent obstructive symptoms Pain related to ostomy Leakage from ostomy Erosion of skin surrounding ostomy Aesthetically unsatisfactory Discomfort Other *	33 (27.27%) 14 (11.57%) 31 (25.62%) 14 (11.57%) 2 (1.65%) 5 (4.13%) 17 (14.05%) 3 (2.48%)	121
Not specified **	2 (1.65%)	
**Type of repair**		
Open with mesh (onlay) Open sugarbaker Open without mesh *** Lap with mesh (keyhole) Lap with mesh (sugarbaker) Lap suture without mesh Pauli (open and laparoscopic)	9 (7.56%) 12 (10.08%) 13 (10.92%) 6 (5.04%) 72 (60.50%) 4 (3.36%) 3 (2.52%)	119
**Urgency of repair**		
Acute surgery Elective	38 (31.93%) 81 (68.07%)	119
**EHS classification**		
1 (≤ 5 cm) 2 (≤ 5 cm plus incisional hernia) 3 (> 5 cm) 4 (> 5 cm plus incisional hernia) NA	79 (66.39%) 13 (10.92%) 18 (15.12%) 3 (2.52%) 6 (5.04%)	119
**Clavien Dindo index score**		
Grade 1 Grade 2 Grade 3a Grade 3b Grade 4 Grade 4a Grade 5	97 (81.51%) 6 (5.04%) 4 (3.36%) 6 (5.04%) 0 (0.00%) 2 (1.68%) 4 (3.36%)	119
**Postoperative surgical complications**		
No Complications, *n* = 70 Seroma, *n* = 13 Pain, *n* = 14 Constipation, *n* = 16 Wound infection, *n* = 5 Deep infection, *n* = 2 Re-operation, *n* = 6 Hematoma around ostomy, *n* = 3 Death, *n* = 4	70 (52.63%) 13 (9.77%) 14 (10.52%) 16 (12.03%) 5 (3.75%) 2 (1.50%) 6 (4.51%) 3 (2.25%) 4 (3%)	133
**Recurrence**		
Recurrence Acute surgery Elective surgery No recurrence Not applicable****	21 (17.64%) 7 (33.33%) 14 (66.67%) 93 (78.15%) 5 (4.2%)	119

*N* = 119, *DM* Diabetes Mellitus, *COPD* Chronic Obstructive Pulmonary Disease, BMI Body Mass Index, *ASA* American Society of Anesthesiologists Classification, *FAP* Familial Adenomatous polyposis, *Obstruction of other urological structures causing hydronephrosis and re-operation with placement of prophylactic mesh and psychological complaints, ** Reason for surgery not stated in journal, ***(stomitransposition or primary suturing), *EHS* European Hernia Society, ****Death or transferred to other hospital

### Univariate analyses

Table 2 provides a detailed overview of the univariate analyses (Chi-squared, unless otherwise indicated) for non-modifiable risk factors against the recurrence rate, whereas Table 3 provides an overview of the univariate analysis for the modifiable factors. We found that younger patients, ≤55 years old, have a significantly higher recurrence rate compared to older patients (*p* = 0.0019). We also found that ileostomy (*p* = 0.0436) and loop ileostomy (*p* = 0.0254) reach significance, indicating that these types of stomas are more susceptible to recurrence. Additionally, reasons for stoma, including colorectal cancer (*p* = 0.0187), IBD (*p* = 0.0314), constipation (*p* = 0.0184), and EHS Classification 1 (*p* = 0.0474) reached significance.

However, in terms of modifiable factors, re-operation (*p* = 0.0554) and Clavien Dindo grade 3b (*p* = 0.0704) both show a tendency.

**Table 2:** Analysis of non-modifiable risk factors

Risk factors	Recurrence rate	Univariate
	(%)	p-value
**Sex**		p = 0.7519 •
Male, n = 49	8 (16.33%)	
Female, n = 70	13 (18.57%)	
**Age**		
≤ 55 years, n = 17	8 (35.29%)	p = 0.0019 (***)
56–65 years, n = 25	6 (24.00%)	p = 0.3778
66–74 years, n = 38	3 (5.26%)	p = 0.0809 (.)
≥ 75 years, n = 39	4 (10.26%)	p = 0.1944
**Smoker**		
Non-smoker, n = 99	16 (16.16%)	p = 0.5322
Current smoker, n = 15	4 (26.6%)	p = 0.4543
Not stated, n = 5	1 (20%)	p = 1
**Co-morbidity**		
Diabetes mellitus, n = 16	4 (25%)	p = 0.5882
COPD, n = 20	4 (20%)	p = 1
Asthma, n = 9	2 (22.22%)	p = 1
**BMI**		
< 18.5, underweight, n = 1	0 (0%)	~
18.5–24.9, normal weight, n = 38	7 (18.42%)	p = 1
25.0–29.9, pre-obesity, n = 45	8 (17.77%)	p = 1
30.0–34.9, Obesity class I, n = 20	5 (25%)	p = 0.5097
35.0–39.9, Obesity class II, n = 5	1 (20%)	p = 1
> 40, Obesity class III, n = 2	0 (0%)	~
**ASA Classification**		
1, n = 4	1 (20%)	p = 1
2, n = 69	14 (20.28%)	p = 0.4598
3, n = 43	6 (13.95%)	p = 0.4878
4, n = 3	0 (0%)	~
5, n = 0	0 (0%)	~
**Type of stoma**		
Colostomy, n = 71	9 (12.67%)	p = 0.0836 • (.)
Ileostomy, n = 35	10 (28.57%)	**p = 0.0436** • (*)
Loop ileostomy, n = 2	2 (100%)	p = 0.0254 (*)
Urological ostomy, n = 11	0 (0%)	~
**Urgency of repair**		p = 0.8794 •
Acute surgery, n = 38	7 (18.42%)	
Elective, n = 81	14 (17.28%)	
**Reason for stoma**		
Colorectal cancer, n = 50	4 (8%)	p = 0.0187 • (*)
Inflammatory bowel disease, n = 23	8 (34.78%)	p = 0.0314 (*)
Benign sigmoid stenosis, n = 5	0 (0%)	~
Intestinal ischemia, n = 3	1 (33.33%)	p = 1
Anal and rectal injuries, n = 5	1 (20%)	p = 1
Fecal incontinence, n = 9	3 (33.33%)	p = 0.3503
Urological cancer, n = 10	0 (0%)	~
Gynecologic cancer, n = 1	0 (0%)	~
Perforated diverticulitis, n = 5	0 (0%)	~
FAP, n = 1	0 (0%)	~
Constipation, n = 7	4 (57.14%)	p = 0.0184 (*)
**Reason for hernia correcting surgery**		
Intestinal obstruction, n = 33	6 (18.18%)	p = 0.9245 •
Recurrent obstructive symptoms, n = 14	4 (28.57%)	p = 0.2509
Pain related to ostomy, n = 31	5 (16.12%)	p = 1
Leakage from ostomy, n = 14	1 (7.14%)	p = 0.4738
Erosion of skin surrounding ostomy, n = 2	1 (50%)	p = 0.3283
Aesthetically unsatisfactory, n = 5	1 (20%)	p = 1
Discomfort, n = 17	3 (17.64%)	p = 1
Other, n = 3	0 (0%)	~
Not specified, n = 2	1 (50%)	p = 0.3333
**EHS Classification**		
1 (≤ 5 cm), n = 79	18 (22.78%)	p = 0.0474 (*)
2 (≤ 5 cm plus incisional hernia), n = 13	1 (7.69%)	p = 0.4658
3 (> 5 cm), n = 18	2 (11.11%)	p = 0.5277
4 (> 5 cm plus incisional hernia), n = 3	0 (0%)	~
NA, n = 6	0 (0%)	~

•Regular chi-square test, but a chi-square Monte Carlo simulated p-value has been used based on 2000 replicates if not otherwise indicated. Significant p-values are written in bold

**Table 3:** Analysis of modifiable risk factors

Risk factors	Recurrence rate	Univariate
	(%)	p-value
**30-day post op surgical complication**		
No Complications, n = 70	10 (14.28%)	p = 0.2503
Seroma, n = 13	2 (15.38%)	p = 1
Pain, n = 14	4 (28.57%)	p = 0.2749
Constipation, n = 16	5 (31.25%)	p = 0.1554
Wound infection, n = 5	1 (20%)	p = 1
Deep infection, n = 2	0 (0%)	~
Re-operation, n = 6	3 (50%)	**p = 0.0554 (.)**
Hematoma around ostomy, n = 3	1 (33.33%)	p = 1
Death, n = 4	NA	NA
**Type of repair**		
Open with mesh (onlay/skorsten), n = 9	0 (0%)	p = 0.2059
Open sugarbaker, n = 12	0 (0%)	~
Open without mesh, n = 13	2 (15.38%)	p = 1
Lap with mesh (keyhole), n = 6	0 (0%)	~
Lap with mesh (sugarbaker), n = 72	14 (19.44%)	p = 0.6152
Lap without mesh, n = 4	2 (50%)	p = 0.1414
Pauli, n = 3	1 (33.33%)	p = 1
**Clavien Dindo index score**		
Grade 1, n = 97	15 (15.46%)	p = 0.1979
Grade 2, n = 6	0 (0%)	~
Grade 3, n = 4	2 (50%)	p = 0.1424
Grade 3b, n = 6	3 (50%)	**p = 0.0704 (.)**
Grade 4a, n = 2	1 (50%)	p = 0.3243
Grade 5, n = 4	0 (0%)	~
**Time of recurrence**		
Within 1 year	15 (71.42%)	
Within 2 years	2 (9.52%)	
Within 3 years	4 (19.04%)	

Significant p-values are written in bold

### Non-modifiable risk factors

We employed LASSO regularization in linear regression using a dataset of 115 observations (4 were excluded due to being outliers) with all factors in Table 2 as predictor variables. Regression results are summarized in Table 4. The selected model yielded 6 non-zero coefficients. The chosen lambda value for regularization was 0.0487 (lambda 1 standard error from the minimum lambda: 0.1235). Performance evaluation indicated a mean squared error of 0.1389. The LASSO procedure effectively identified significant predictors, achieving sparsity by shrinking several coefficients to zero. The candidate variables were selected as predictors for our GLM model below.

We computed a GLM with recurrence rate as dependent variable and the LASSO-qualified variables as independent variables. These were: age, diabetes, loop ileostomy, inflammatory bowel disease, fecal incontinence, EHS classification, and constipation (reason for stoma). The first model resulted in extreme estimates for the loop ileostomy, so we suspected that multicollinearity was interfering with the quality of the fitted model. By removing loop ileostomy as a factor, we ran a second model and compared it to the first one. As the second model (without loop ileostomy) proved to perform better (BIC: 112.13 < 114.59. Log likelihood: -39.46 < 38.31), we went with that model. The GLM revealed, similar to the univariate analysis, that younger patients had a significantly higher chance for recurrence (OR = -0.052, SE = 0.02, *p* = 0.0292), and that only patients with DM (3 levels: yes, no, unknown) had a significantly greater chance for recurrence (OR = 1.727, SE = 0.83, *p* = 0.0386). We also observed that IBD (OR = 1.604, SE = 0.72, *p* = 0.0260), fecal incontinence (OR = 2.455, SE = 0.92, *p* = 0.0078) and constipation (OR = 2.719, SE = 0.99, *p* = 0.0060) all significantly affected the recurrence rate. In other words, we observe an age-related decrease in recurrence rates and that patients with either fecal incontinence or constipation *prior to the operation* have a 23.29% and 30.33% chance to experience recurrence, respectively. See Table 4 for confidence intervals.

To be sure, this model suggests that for a 35-year-old patient with fecal incontinence has 70.22% chance for recurrence, compared to one who has both fecal incontinence and diabetes where chances are 83.98%.

$$\begin{aligned}\:0.513\:+\:\left(35\:*\:-0.052\right)+\:\left(1\:*\:1.727\right)+&\:\left(0\:*\:1.604\right)+\cr\quad\:\left(0\:*\:2.455\right)+\:\left(1\:*\:2.719\right)=3.139\end{aligned}$$

$$\:\frac{{e}^{0.513}*3.139}{1+\left({e}^{0.513}*3.139\right)}=83.98\%\:for\:recurrence$$

That’s an increase of 13% simply due to DM. However, this should be interpreted with caution given the low number of observations.

**Table 4:** Non-modifiable GLM estimates

Variables	Estimates (odds ratio)	95% CI	*P* value	Chances (odds/1 + odds)
(Intercept)	0.513	[-2.70 3.73]	0.7543	
Age	-0.052	[-0.09 -0.005]	0.0292 *	-9.51%
Diabetes-Yes	1.727	[0.09 3.36]	0.0386 *	74.25%
Inflammatory bowel disease	1.604	[0.19 3.01]	0.0260 *	72.81%
Fecal incontinence	2.455	[0.64 4.26]	0.0078 **	80.39%
Constipation	2.719	[0.77 4.66]	0.0060 **	81.95%
**Factors included but not retained at the p value of < 0.1**:				
Diabetes-Unknown, *p* = 0.9150				

This table represents the final model after subjecting selected non-modifiable factors to a LASSO regression. Candidate variables were then transferred to this GLM regression. The chances should be read, for instance for age, as “for every increase in years of a patient, chances decrease by 9.51% for recurrence.” *p* < 0.05 (*). *p* < 0.01 (**)

### Modifiable risk factors

In a similar fashion, we employed the LASSO technique using the same dataset, however, with all factors in Table 3 as predictor variables. The LASSO regression analysis minimized all coefficient estimates to zero, indicating that none of the variables demonstrated sufficient predictive strength to be retained. As a result, no variables were selected for further analysis or consideration in the model.

## Discussion

The main finding in this study is that the recurrence after primary PSH surgery is as low as 12.60% within the first year, and up to 19.04% within three years. This is less than reported in the literature [3, 5, 6]. We hypothesize that this low recurrence is due to centralized care, ensuring standardized treatment from referral. The standard treatment involves clinical assessment and pre-operative Computed Tomography (CT) scans, which are collaboratively reviewed by experienced surgeons, optimizing surgical planning and patient selection. Hernia surgery is performed by experienced hernia surgeons and likely contribute to improved outcomes and lower recurrence rates. This comprehensive approach minimizes diagnostic variability, ensures consensus on indications and standardizes type of surgical repair at the center, ultimately enhancing overall patient care.

Twenty-one patients experienced recurrence of their parastomal hernia, with seven undergoing emergent surgery and fourteen opting for elective surgery. In the univariate analysis, we observed no significant difference between these groups (*p* = 0.8794). Additionally, emergent surgery did not appear as a significant predictor in the LASSO algorithm, suggesting its lack of predictive value for recurrence. We attribute this outcome to the established practice of avoiding emergency hernia reconstruction at the center. Patients categorized with an emergent surgery means that the patient underwent a two-stage procedure within the same hospital admission. Initially the emergency operation would be reduction of the hernia either by laparoscopic or open approach followed by admission to the department and pre-operative optimization. The patient then undergoes evaluation by a hernia surgeon, who plans and performs the hernia correcting surgery if deemed appropriate, as it is known that surgeon volume is a big factor for better results [19].

83% of patients were non-smokers as there is a strict policy at the department prohibiting smoking before elective surgery. We suggest that this policy partly accounts for the low incidence and severity of complications, with 81.51% experiencing Clavien-Dindo grade 1, and only four and six patients classified as grade 3a and 3b, respectively. Constipation and pain are the two most common complications. Around 60% of patients have undergone laparoscopic sugarbaker and this operation is known to cause postoperative pain due to tacks attached to the abdominal wall. Constipation is also a known postoperative factor after abdominal surgery and therefore expected. In the department, there is a strict regime regarding lack of bowel movement and this is assed every day during admission. Seroma, to the extent where it was clinically palpable or verified on a CT scan, was also one of the most frequent complications. It is possible that seroma as a clinically palpable bulging around stoma, could have been a recurrent PSH, but disregarded as seroma by the surgeon examining the patient in the cases where no CT scan was performed. This could potentially underestimate the incidence of recurrence. As mentioned, a majority of patients have undergone laparoscopic sugarbaker and this type of repair leaves the hernia sac in place during the operation. This results in a dead space that is susceptible to serous fluid accumulation, increasing the likelihood of seroma formation.

Through both the univariate and multivariate analyses we observe that inflammatory bowel disease ( significantly increased the risk of recurrence. IBD patients are predisposed to hernia formation, as they have a history of use of immunomodulatory drugs and often a history of multiple abdominal operations, contributing to a fascial weakening of the abdominal wall [20]. Moreover, the univariate analysis revealed loop ileostomy and ileostomy as significant factors for recurrence and we can speculate that patients with IBD indeed is part of this group, underlining the risk associated with operating on IBD patients. Aligning with the literature, we also find that patients with DM have an increased chance for recurrence [21].

It is an acceptable concept that a defect in the abdominal wall will increase with a rise in the Intra-Abdominal Pressure (IAP) [22]. We found that patients with symptoms of constipation pre-operatively was in a significantly higher risk of recurrence, which is supported by the pathophysiology of an increased intraluminal volume causing an increased IAP. Efforts should be made to reduce symptoms of constipation before planning for hernia correcting surgery. Furthermore, we found that colorectal cancer and obstipation as reason for creating of stoma to begin with, has a significant influence on the risk of recurrence. We attribute this to the fact that pre-operative optimizing of constipation is usually not as intense as other morbidities such as heart disease, high BMI etc.

Our statistical model estimated that age was a significant factor with a negative estimate, meaning that either the model has an underrepresentation of younger patients, biasing the model to estimate them as more prone to recurrence, or that the younger group has more comorbidities resulting in increased chances for recurrence. We interpret these results to suggest that younger patients were likely more severely ill, making them more susceptible to recurrence compared to older patients. Therefore, the model’s findings should not be taken at face value but rather be considered in the context of factors such as diabetes, IBD, fecal incontinence, or constipation as these factors significantly increase chances for recurrence, which, in turn, may require a different approach. Further research with a larger and more diverse sample is necessary to gain a more comprehensive understanding of these outcomes.

In Table 3, we see that the highest rate of recurrence is to be found in the group of patients that have been repaired laparoscopically without mesh (50%). The sugarbaker method has a recurrence of 19.44%, but is also the most frequently performed operation. The other group of patients with the highest percentage of recurrence is when the PSH was an open repair with no mesh (15.38%). The group of patients that underwent hernia repair with Pauli, experienced a recurrence of approximately 33%. No recurrence was observed within the other groups that were repaired with mesh involving open repair with mesh, open sugarbaker, and keyhole repair.

Several studies have demonstrated that mesh repair significantly reduces recurrence rates compared to suture repair in parastomal hernia cases, where recurrence is described up to 69.4% [23–25]. The biomechanics of suture repairs can lead to high tension on the suture line, increasing the risk of sutures pulling through tissue and reopening the hernia defect. Repair with mesh on the other hand provide structural reinforcement, distributing tension more evenly and strengthening the abdominal wall, thereby minimizing the risk of recurrence [26]. Furthermore, suture repairs rely entirely on the body’s natural healing processes, which may be insufficient in cases of poor tissue quality or elevated abdominal pressure [27]. This also underlines the importance of pre- and post-operative treatment of constipation, which our results indicate can be a reason for parastomal hernia recurrence.

We used a follow-up period of minimum 3.5 years, as we were restricted when viewing the patients’ medical journals due to GDPR rules. In our study, we found that the incidence of PHS recurrence reduces each year after parastomal hernia surgery, given the negative beta-estimate in our GLM. This is in contrast to other studies indicating an increase in the following years [9]. However, at the third year, we observe an increase, which suggests a longer follow-up period for future research.

EHS classification 1 is significant for recurrence. This might be due to overrepresentation. The finding that Clavien Dindo grade 3b is associated with an increased risk of parastomal hernia recurrence is actually an anticipated result, given the severity of complications classified at this level.

We observe that we have more significant variables/predictors for pre-operative factors compared to post-operative factors, which speaks to the fact that pre-operative factors are better predictors for experiencing recurrence than post-operative factors. In other words, post-operative observations may not be good indicators compared to pre-operative predictors.

The study is limited due to its retrospective nature. The sample size is small and the number of patients undergoing different types of hernia repair is not large enough to draw strong conclusions about the superiority or inferiority of any technique. However, the strength of this study, is that the population is well defined. The medical records have been thoroughly examined and details recorded.

## Conclusion

This study shows that after nationwide centralization of hernia repair in Denmark, the recurrence rate of parastomal hernia after primary repair is low at the Hernia Center at Horsens Hospital compared to the current literature. When accounting for multiple risk factors, our study finds that good pre-operative predictors include age,DMd, IBD, constipation, and fecal incontinence. However, when considered separately, our study finds that again age, IBD, and constipation along with ileostomy, loop ileostomy, and colorectal cancer are good indicators. For post-operative variables, there we no good indicators when considering multiple risk factors simultaneously, but we find that re-operation, EHS classification 1, and Clavien Dindo Grade 3b are good predictors for recurrence when considered univariately.

The results are limited to the Danish population and cannot be generalized to an international level. However, these insights on risk factors that affect the development of parastomal hernia recurrence suggest guidelines when assessing patients and play a role in early prevention of recurrence.

## Data Availability

Data are not available for public access, due to the presence of confidential patient information.

## Declarations

Access to data has been accepted by all of the hospitals in the Central Jutland Region and the respective hospital directors.
